# Residual lesions in patients undergoing microsurgical clipping of cerebral aneurysms in a reference university hospital

**DOI:** 10.6061/clinics/2020/e1973

**Published:** 2020-11-02

**Authors:** Guilherme Brasileiro de Aguiar, Matheus Kohama Kormanski, Carolina Junqueira Tavares Corrêa, Andrew Vinícius de Souza Batista, Mario Luiz Marques Conti, José Carlos Esteves Veiga

**Affiliations:** I Faculdade de Ciencias Medicas da Santa Casa de Sao Paulo (FMSCSP), SP, BR

**Keywords:** Intracranial Aneurysm, Microsurgical Clipping, Cerebral Angiography, Remaining Aneurysm

## Abstract

**OBJECTIVES:**

This study aimed to analyze the incidence and epidemiological, angiographic, and surgical aspects associated with incomplete clipping of brain aneurysms in a cohort of patients undergoing microsurgical treatment.

**METHODS:**

The medical record data of patients who underwent microsurgery for cerebral aneurysm treatment and postoperative digital subtraction angiography, treated at the same teaching hospital between 2014 and 2019, were retrospectively analyzed. The studied variables involved epidemiological and clinical data, as well as neurological status and findings on neuroimaging. The time elapsed between hemorrhage and microsurgical treatment, data on the neurosurgical procedure employed for aneurysm occlusion, and factors associated with the treated aneurysm, specifically location and size, were also evaluated.

**RESULTS:**

One hundred and seventeen patients were submitted to 139 neurosurgical procedures, in which 167 aneurysms were clipped. The overall rate of residual injury was 23%. Smoking (odds ratio [OR]: 3.38, 95% confidence interval [CI95%]: 1.372-8.300, *p*=0.008), lesion size >10 mm (OR: 5.136, CI95%: 2.240-11.779, *p*<0.001) and surgery duration >6 h (OR: 8.667, CI95%: 2.713-27.681, *p*<0.001) were found to significantly impact incomplete aneurysm occlusion in the univariate analyses.

**CONCLUSION:**

Incomplete microsurgical aneurysm occlusion is associated with aneurysm size, complexity, and current smoking status. Currently, there is no consensus on postoperative assessment of clipped aneurysms, hindering the correct assessment of treatment outcomes.

## INTRODUCTION

Major advances in the diagnosis and management of cerebral aneurysms have been made in the past 30 years (1). Generally, the ultimate goal of aneurysm treatment is to achieve complete occlusion while avoiding rupture or rebleeding (2) and preserving the patency of adjacent arteries. This treatment can be performed using microsurgical techniques or the endovascular approach (1-3).

Recent studies attribute greater rates of complete occlusion of aneurysms to microsurgery (4-6). Although microsurgical clipping seems to be the most effective and durable treatment (2,7), most studies that investigate this treatment have no postoperative control group to confirm this theory.

Despite the new technologies used in microsurgery of intracranial aneurysms, such as intraoperative indocyanine green or fluorescein videoangiography (8) and intraoperative micro-Doppler examination, the percentage of cases with incomplete occlusion of lesions remains relatively constant (8). However, it is important to recognize and follow-up partially treated lesions considering the associated risk of future hemorrhages.

Therefore, identifying factors that may be associated with incomplete treatment of cerebral aneurysms is paramount for optimal treatment planning. The objective of the present study was to analyze the incidence, as well as the epidemiological, angiographic, and surgical aspects associated with incomplete occlusion of aneurysms in a cohort of patients that underwent microsurgical treatment.

## MATERIAL AND METHODS

A retrospective study was performed of the medical records of patients who underwent microsurgical treatment of ruptured and unruptured cerebral aneurysms and postoperative digital subtraction angiography (DSA) at the Division of Neurosurgery of the Irmandade da Santa Casa de Misericórdia de São Paulo between 2014 and 2019. This study was approved by the Research Ethics Committee at our Institution (2.647.409) and patients signed the informed consent for participation in the study. Patients with missing information from medical records or those who, for any reason, refused to take part in the study were excluded from the cohort.

The following variables were studied: epidemiological data (age, sex, comorbidities, smoking status), neurological status (scores on the Glasgow Coma Scale, Hunt & Hess scale, and presence of clinical and angiographic vasospasm and hydrocephalus—diagnosed by cranial tomography), data about the neurosurgical procedure used for aneurysm clipping, in addition to factors associated with the treated aneurysm, specifically location, and size.

For the classification of residual lesions, we used the Sindou classification system (9). This scale classifies the morphology of aneurysm remnants into five grades: I - <50% of neck size; II - >50% of neck size; III - residual lobe of a multi-lobulated sac; IV - residual portion of sac <75% of aneurysm size; and V - residual portion of sac >75% of aneurysm size.

### Statistical analysis

All relevant data were tabulated and organized for the elaboration of exploratory descriptive statistics. For inferential analysis, Fisher's exact association test was used. The odds ratio (OR) was calculated using binary logistic regression with the IBM^®^ Software SPSS, version 22, for those variables reaching significance at the level of 5%. Data from ruptured and unruptured aneurysms were pooled for statistical analyses.

## RESULTS

One hundred and seventeen patients were submitted for the microsurgical treatment of ruptured and unruptured cerebral aneurysms and cerebral angiography for postoperative evaluation at our hospital. These patients were submitted to 139 neurosurgical procedures, in which 167 aneurysms were clipped. Patients were predominantly female (75.2%), and age ranged from 20 to 59 years. The vast majority of patients (74%) had hypertension and 58% were current smokers (Table 1). Among 139 surgeries, 42 (30.2%) were performed in patients without subarachnoid hemorrhage. Three hundred and seventeen additional surgeries for aneurysm clipping were excluded from our analysis due to the lack of postoperative DSA, since these patients had unfavorable outcomes or were transferred to secondary hospitals and lost to follow-up.

Upon admission, the Hunt & Hess grade was generally low (range, 1-3) for 95.9% of the patients in the ruptured aneurysm group (Table 2). In contrast, the Fisher grade was generally poor (66%) for these patients, mainly represented by patients with Fisher grade 4 (37%). Vasospasm was documented in 25.8%, and radiological hydrocephalus occurred in 17.5% of 97 surgeries for subarachnoid hemorrhage (Table 2). Most patients had only one aneurysm clipped per procedure (82%) and underwent surgery during morning shifts via elective surgery (Table 3). Furthermore, surgeries lasted generally between 4 and 6 h (72.2%) (Table 3).

The overall rate of residual lesion was 23% (38 of 167 aneurysms). Grade I aneurysm residuals were found in 13 aneurysms (34%), Grade II in 9 aneurysms (24%), Grade III in 10 aneurysms (26%), Grade IV in 2 aneurysms (5%), and Grade V in 4 aneurysms (10%). For the subgroup of patients who underwent surgery for subarachnoid hemorrhage, residual lesions were detected in approximately 26% of patients through postoperative DSA. Conversely, aneurysms smaller than 10 mm had residual lesions in about 16.3% of patients. Among the residual lesions, nine underwent reoperation either for being classified into Sindou groups III, IV, or V, or because the residual necks enlarged during follow-up (23 months, range 8-53 months). No residual necks were documented after retreatment.

The pooled statistical analyses revealed the association between incomplete clipping and current smoking (*p*=0.008), as well as surgery duration greater than 6 h (*p*<0.001) and aneurysm size greater than 10 mm (*p*<0.001). No association was found between location, multiplicity or ruptured/unruptured aneurysm, and incomplete treatment (Table 4). Univariate analyses revealed an increased risk of residual aneurysms on postoperative DSA for current smokers (OR: 3.38, 95% confidence interval [CI95%]: 1.372-8.300, *p*=0.008). In addition, risk analyses showed that patients whose surgical time exceeded 6 h had almost a nine times greater probability of having residual lesions than patients whose surgery took less than 4 h (OR: 8.667, CI95%: 2.713-27.681, *p*<0.001). Lesion size was also associated (*p*<0.001) with residual aneurysms, in which lesions >10 mm were five times more likely to be partially occluded (OR: 5.36, CI95%: 2.240-11.779, *p*<0.001).

## DISCUSSION

Since its description by Walter Dandy in 1938, microsurgical occlusion of cerebral aneurysms using metal clips has been employed as the definitive treatment of this condition (2,6,10). The emergence and refinement of endovascular intervention techniques for treating cerebral aneurysms have led to the inevitable comparison of the efficacy of the two modalities. Although the goal of treatment is always complete definitive aneurysm occlusion, this is not always achieved (10) in either approach. Consequently, the occurrence of residual aneurysms is not uncommon. The existence of a residual aneurysm after microsurgery is defined as a small segment at the base of a clip still filled with contrast material (11). This definition is adopted by most authors (8,11), and thus was used for the purposes of this study. Some studies, however, stipulate a minimum size of 1 mm as the criteria for defining a residual aneurysm (11).

In general, the data published in the literature estimated incomplete occlusion rates after microsurgery of 1.6-42% (8,10,11). In the present casuistic, 23% of aneurysms had residual lesions, which is within the range reported. Additionally, the rate (8.2%) among a group of experienced surgeons was not low, as shown by David et al. (12) in a series of 147 operated aneurysms.

Unlike aneurysms submitted to endovascular treatment, which are exhaustively followed and assessed for complete occlusion and potential recurrence, cases submitted to microsurgical treatment are followed based on the intuitive attitude of the surgeon. In these cases, postoperative angiography is often lacking, thereby precluding detection of subsequent residual lesions (7,11). It is widely recognized that few surgeons perform postoperative control of clipped aneurysms (12).

More recently, with the increasing use of endovascular techniques, postoperative control has become obligatory in all techniques (9,12). In this regard, the International Subarachnoid Aneurysm Trial (13) showed residual lesion rates of up to 26% in aneurysms treated using the endovascular approach and approximately 12% in cases managed with microsurgery (1,13). However, the rate of aneurysm occlusion after rebleeding was not considered. Overall, it is believed that residual lesions are identified in 4-19% of cases treated for intracranial aneurysms (8).

More recently, the Cerebral Aneurysm Rerupture After Treatment (CARAT) study (5) highlighted that there is a percentage of aneurysms not fully occluded in both therapeutic modalities; however, the rate of complete occlusion associated with microsurgery is higher (4,5). Curiously, in the same CARAT study, patients with complete occlusion of aneurysms were younger, had lower rates of chronic pulmonary disease, and fewer posterior circulation aneurysms than patients with incomplete occlusion. In the current patient sample, these aspects were not associated with aneurysm occlusion rates. According to the cited study (4), incidence of intraoperative complications was no greater for microsurgical than for endovascular techniques, when complete occlusion of aneurysms was achieved. The fact that significant advancements in the technology involved in microsurgical and endovascular management of cerebral aneurysms has rendered treatment safer (1) further reinforces the notion that complete occlusion is the ultimate goal of cerebral aneurysm treatment.

However, there has been a notable increase in the rate of residual lesions in case series published over the past few years, and this is largely because of the greater postoperative follow-up of operated lesions (10). Nevertheless, both techniques (microsurgery and embolization) have proven safe and effective for the management of aneurysms (1), with a greater tendency for use of endovascular treatment in recent years (1), especially in Europe.

Akin to the present study, Kim et al. (11) also sought to associate clinical characteristics (systemic arterial hypertension, diabetes mellitus, and dyslipidemia) with the occurrence of residual lesions. Similarly, they failed to find a significant association. The rationale for seeking an association is linked to the fact that atherosclerotic lesion can cause stiffening and shrinking of the vessel wall, hindering the application of the clip to the aneurysm. This was the explanation provided by Dellaretti et al. (8) for the higher incidence of residual lesions in older patients. In the present study, the majority of the casuistic presented no dyslipidemia and no association with this factor; additionally, the presence of residual lesions was evident. Only current smoking was associated with the occurrence of residual lesions, possibly because of its effects on the artery walls. Except for this aspect, involvement of perforating arteries at the deep base of the aneurysm contributes to incomplete inclusion (8,11).

Given the clear need for long-term follow-up of operated aneurysms, these lesions should be controlled by using imaging. Although many studies have employed computed tomography angiography and magnetic resonance angiography for follow-up, DSA remains the method offering best accuracy and specificity for diagnosing residual lesions after microsurgical treatment of cerebral aneurysms (2,14,15). DSA yields information on clip position, presence of residual lesions, occlusion, or stenosis of adjacent vessels, and the presence of other untreated aneurysms (12).

The fact that residual aneurysms require attention and care has been recognized since the 1960s, following the studies of Drake et al. (2,9,16,17). Besides posing a risk of bleeding, some authors have suggested that residual lesions can evolve with spontaneous thromboses (12,16). Nevertheless, postoperative control is not performed in most cases (16). In the study by Feuerberg et al. (16), some small residual aneurysms evolved with spontaneous thrombosis; however, this should not be considered the natural evolution of residual lesions.

The heightened concern over partially treated aneurysms is related to the risk of rupture. According to Johnston et al. (4), there is a 24.5% risk of rupture of poorly occluded aneurysms within 1 year of surgery. This risk is similar to that estimated for rebleeding in untreated aneurysms (4). Other authors cite higher risks ranging from 0.5-1.9% per year (1,10,12). In addition, the probability of rupture of a residual lesion is directly related to the degree of occlusion of the aneurysm (4,5) and, in most cases, occurs within the first year after treatment (4), justifying early control examinations.

Aneurysms treated using embolization have a greater propensity for incomplete occlusion and, thus, represent most cases of rebleeding that require retreatment (4-6,10). The need for retreatment in cases of aneurysms previously submitted to surgical treatment using microsurgery and presenting residual lesions is estimated at 1.5% of patients (7). Although the percentage of cases of residual aneurysms is higher after endovascular treatment, residual lesions after microsurgery appear to have greater risk of bleeding (1). Moreover, residual aneurysms can increase in size or lead to symptoms as a result of the mass effect (1). Increases in the diameter of residual lesions have been reported in up to 50% of cases (1). In our series, nine aneurysms were retreated, because of either the enlargement during follow-up or the large size of the residual lesion. Retreatment for an additional bleeding episode was performed in only one case. Treatment of residual aneurysms is challenging *per se*, given the changes in normal morphology of the lesion and previous manipulation (1). However, these can be safely and effectively resolved by either endovascular or microsurgical management (1).

Another aspect involved in the postoperative assessment of cerebral aneurysms is the risk of recurrence. Although endovascular treatment is more prone to recurrence, this risk also exists after microsurgical management (6,18). The aneurysm can recur through the development of residual lesions or stem from an angiographically cured aneurysm (6,12,18); in the latter case, possible causes include clip slippage and vascular weakening associated with local hemodynamic stress (6,12,17). Typically, the risk of aneurysm recurrence associated with incomplete occlusion by clipping is around 2.9% per year, while the risk of hemorrhage is approximately 1.5% per year (12). Notably, in the present study, both treatment durability recurrent or “de novo” lesions were not assessed, given that the authors sought to detect residual, as opposed to recurrent, lesions. There is no consensus in the literature on the ideal follow-up time for these cases (10); however, some reports suggest performing angiography within the first post-surgical month and again at a later date, even if the first exam shows cure of the aneurysm (12).

Few studies have investigated the risk factors for occurrence of residual lesions after microsurgical treatment (10); instead, these studies have focused on lesion location, size, and morphology (10,11). In the present study, a number of different variables were analyzed to determine associations between aspects inherent to the surgical procedure and failure to achieve complete occlusion. Our findings revealed the association to lasting surgeries (>6 h). This finding, however, cannot be definitively associated with residual aneurysms, since more complex lesions typically demand longer procedures. Although not explored in the present study, occurrence of residual lesions has been associated with a higher number of clips used, as shown by Dellaretti et al. (8) This may also be associated with a greater complexity of the aneurysms treated, in addition to the clip placement based on the intuition of the operating neurosurgeon (11).

Other authors have attempted to establish a link between the presence of residual aneurysm and specific variables, such as the presence of acute or previous subarachnoid hemorrhage, multiple aneurysms, or start time of surgery (10). These same variables were assessed in the present study, and no statistically significant results were found to support this association.

Numerous publications are available on the characteristics of surgically-treated aneurysms with residual necks. Most of these reports seek to associate lesion size and location with risk of incomplete treatment (1,3,8,10,11,16,19). In general, with regard to site, aneurysms in ophthalmic, anterior and posterior communicating arteries, as well as those located in the posterior circulation, appear to have higher rates of incomplete occlusion (10). An analysis that reported an association between residual lesions and site after endovascular treatment, in addition to the locations outlined above, also included intracavernous lesions (1). In the analysis of the present cohort, there was no influence of location on the occurrence of residual lesions in different artery segments or anterior and posterior circulations. This result corroborates the findings of other authors (8,11).

Although no statistically significant relationship was reported, some studies continue to associate aneurysm location with incomplete treatment (1,2,3,15). In the study of Jabbarli et al. (10), the incidence of residual aneurysm was 18.2% of the 659 aneurysms studied, which was lower than the 23% observed in the present study. In the study of Jabbarli et al. (10), the majority of aneurysm remnants involved the middle cerebral artery, although these residual lesions proved the least likely to require retreatment. In the view of other authors, however, the greatest risk of residual lesions occurs for lesions of the anterior (11,16) or posterior (19) communicating arteries.

Regarding aneurysm size, the rate of residual aneurysms rises based on the size of the initial lesion (8,9,11,19) or neck size (11). These correlations seem logical, given that treatment of a larger the lesion is more complex compared to a smaller lesion. A statistically significant association between aneurysm size and incomplete treatment was found in the present study, where initial lesions >10 mm had a five-fold greater probability of residual lesions. This finding is similar to results reported by Jabbarli et al. (10), who confirmed intraoperative rupture and aneurysm size >12 mm as the only independent variables.

Recently, amid shifts in the conception and need for comparing cerebral aneurysm treatment approaches, different technologies have been employed to improve outcomes (3,8,19). The old idea that the simple opening of the aneurysm after clipping would be proof of its complete occlusion is no longer applicable (16), despite recognizing that the deep base of the aneurysm is the most common rupture site, and its incomplete occlusion, excluding the base, will reduce the rate of rebleeding (16). Drawing on these premises, a variety of different devices have become part of routine microsurgery of cerebral aneurysms, allowing the incidence of incomplete treatment to be reduced (20). According to Chiang et al. (3), the use of intraoperative angiography can allow clip repositioning in 6.5% of patients, reducing the number of residual necks. Similarly, Alexander et al. (19), using the same intraoperative angiography, repositioned clips in 11% of cases, for both occlusion of adjacent vessels and incomplete aneurysm occlusion. An alternative is the use of intraoperative videoangiography together with indocyanine or fluorescein, which also helps identify incomplete occlusion of aneurysms during the transoperative stage (8,20). In spite of the use of these innovative devices, many case series continue to report high rates of aneurysm remnants (8,10).

Regarding residual lesions, a systematic review by Kotowski et al. (2) reported a lack of consensus on a definition or classification, hampering documentation and planning of treatment. The main assessment scales are those devised by Sindou et al. (9), David et al. (12), Raymond et al. (21), and Roy et al. (22). None of these instruments are readily reproducible or validated, and interobserver discrepancy is high (2). However, for its wider range of options, the Sindou classification (9) is the most commonly used in publications. Although a clear association between rupture risk and Sindou (9) Grade III, IV, and V aneurysms exists, there is no data substantiating the distribution of frequency of each subtype. Yet, no publication supports the most frequent types of residual injuries. In general, it is believed that Sindou types I and II—which are benign lesions—are the most frequently found. Such findings may represent an attempt to reconstruct the aneurysmal neck with the intent of not stenosing the carrier artery at the time of clipping. Our results mostly show low grade residual lesions (Sindou I-II).

This study had some important limitations. First, approximately two thirds of our patients could not be included in the analysis due to lack of postoperative DSA. Patients who died, were transferred, or lost to follow-up were not evaluated based on our exclusion criteria. This limits generalizability of our results, but gives further support to the notion that even in “better” surgical scenarios, residual lesions are more frequent than previously expected. Second, the retrospective design of our study carries the limitations of referral and selection bias. In addition, this study reflects a single center experience involving several experienced and young neurosurgeons.

## CONCLUSIONS

Routine postoperative angiographic assessment should be carried out in all patients submitted to microsurgical treatment of cerebral aneurysms. DSA can accurately detect residual lesions and contribute to the decision-making process for retreatment. Incomplete microsurgical aneurysm occlusion is associated with aneurysm size, complexity, and current smoking. Residual lesions occurred in 23% of our series, which were mostly low grade. Retreatment was necessary in approximately 25% of all residuals due to higher-grade aneurysm remnants or aneurysm regrowth. Our results showed low risk of rebleeding in the intermediate-term follow-up. Currently, there is no consensus on the postoperative assessment of aneurysms treated using the microsurgical technique, hindering the correct assessment of treatment outcomes.

## AUTHOR CONTRIBUTIONS

Aguiar GB contributed with conceptualization, data collection, and writing the article. Kormanski MK, correa CJT and batista AVS contributed in data collection, patient assessment, and compiling the article results. Conti MLM contributed in reviewing the manuscript and case assessment, and content improvement. Veiga JCE contributed in reviewing the manuscript and content improvement.

All authors accept full responsibility for all aspects of the research study, ensuring that queries about the accuracy or integrity of any part of the study can be adequately investigated and resolved, and all authors were sufficiently involved in the study to take public responsibility for appropriated parts.

## Figures and Tables

**Table 1:** Analysis of the association of demographic and clinical profiles with the presence of residual lesion in patients who underwent control cerebral angiography after microsurgical treatment of cerebral aneurysm at the HC-ISCMSP between 2014 and 2019. n:117. São Paulo (SP).

	Residual Lesion		
	Yes	No	
	n (%)	n (%)	*p*-value^1^
Demografic Profile			
Sex			0.641
Female	25 (21.37)	63 (53.85)	
Male	10 (8.55)	19 (16.23)	
Age Group			0.486
≤19 years (Young)	0 (0.00)	1 (0.85)	
20|-|59 years (Adult)	32 (27.35)	67 (57.26)	
≥60 years (Elderly)	3 (2.56)	14 (11.98)	
Clinical Profile			
SAH			0.363
Yes	24 (20.51)	63 (53.85)	
No	11 (9.40)	19 (16.24)	
DM			0.345
Yes	2 (1.71)	10 (8.55)	
No	33 (28.20)	72 (61.54)	
Smoking			**0.008**
Yes	27 (23.08)	41 (35.04)	
No	8 (6.84)	41 (35.04)	
Dyslipidemia			0.502
Yes	2 (1.71)	9 (7.69)	
No	33 (28.21)	73 (62.39)	
Obesity/Overweight			0.177
Yes	0 (0.00)	6 (5.13)	
No	35 (29.91)	76 (64.96)	
HIV			1.000
Yes	1 (0.85)	1 (0.85)	
No	34 (29.06)	81 (69.24)	
Alcoholism			0.731
Yes	4 (3.42)	7 (5.98)	
No	31 (26.50)	75 (64.10)	

Source: The Medical and Statistical File Service (SAME) of the Irmandade da Santa Casa de Misericórdia de São Paulo (ISCMSP).

1 Fisher’s Exact Test, at 5% significance level.

Legend: SAH - systemic arterial hypertension; DM: diabetes mellitus; HIV: infection by human immunodeficiency virus.

**Table 2:** Analysis of the association of neurological status, imaging findings, and time elapsed to neurosurgical treatment in individuals who underwent microsurgical treatment of cerebral aneurysm after presenting aneurysmal subarachnoid hemorrhage with presence of residual lesion in patients who underwent control cerebral angiography after microsurgical treatment of cerebral aneurysm at the HC-ISCMSP between 2014 and 2019. n:97. São Paulo (SP).

	Residual Lesion		
	Yes	No	
	n (%)	n (%)	*p*-value^1^
Neurological Status			1.000
3-8 Glasgow	0 (0.00)	2 (2.06)	
9-13 Glasgow	1 (1.03)	5 (5.15)	
14-15 Glasgow	24 (27.74)	65 (67.01)	
Hunt & Hess			0.453
Hunt &Hess 1	5 (5.15)	10 (10.31)	
Hunt &Hess 2	17 (17.53)	42 (43.30)	
Hunt &Hess 3	3 (3.09)	16 (16.49)	
Hunt &Hess 4	0 (0.00)	4 (4.12)	
Time Elapsed between Rupture and Surgery			0.693
0-2 days	3 (3.13)	8 (8.33)	
2-7 days	6 (6.25)	21 (21.88)	
7-30 days	7 (7.29)	25 (26.04)	
≥30 days	9 (9.38)	17 (17.71)	
Vasospasm			0.289
No	21 (21.65)	51 (52.58)	
Yes	4 (4.12)	21 (21.65)	
Hydrocephalus			0.223
No	23 (23.71)	57 (58.76)	
Yes	2 (2.06)	15 (15.46)	
Fisher Scale			0.865
Fisher 1	1 (1.03)	4 (4.12)	
Fisher 2	9 (9.28)	19 (19.59)	
Fisher 3	7 (7.22)	24 (24.74)	
Fisher 4	8 (8.25)	25 (25.77)	

Source: the SAME of the ISCMSP - Discipline of Neurosurgery.

1 Fisher’s Exact Test, at 5% significance level.

**Table 3:** Analysis of the association between presence of residual lesion and variables pertaining to procedure for microsurgical treatment of cerebral aneurysm in patients who underwent control cerebral angiography at the HC-ISCMSP between 2014 and 2019. n:139. São Paulo (SP).

	Residual Lesion		
	Yes	No	
	n (%)	n (%)	*p*-value^1^
Operated after Hemorrhage			0.297
No	8 (5.76)	34 (24.46)	
Yes	27 (19.42)	70 (50.36)	
No. of Aneurysms Clipped in the Procedure			0.238
1	26 (18.71)	88 (63.31)	
2	8 (5.76)	15 (10.79)	
≥3	1 (0.72)	1 (0.72)	
Operation Shift			0.164
Morning	25 (17.99)	55 (39.57)	
Afternoon	7 (5.04)	37 (26.62)	
Night	3 (2.16)	12 (8.63)	
Elective/Urgent			0.692
Elective	19 (13.67)	62 (44.60)	
Urgent	16 (11.51)	42 (30.22)	
Duration			**<0.001**
<4 h	7 (5.04)	39 (28.06)	
4-6 h	14 (10.07)	56 (40.29)	
>6 h	14 (10.07)	9 (6.47)	

Source: the SAME of the ISCMSP - Discipline of Neurosurgery.

1 Fisher’s Exact Test, at 5% significance level.

**Table 4:** **Analysis** of the association between characteristics of clipped aneurysms and presence of residual lesion in patients who underwent control cerebral angiography after microsurgical treatment of cerebral aneurysm at the HC-ISCMSP between 2014 and 2019. n:167. São Paulo (SP).

	Residual Lesion		
	Yes	No	
	n (%)	n (%)	*p*-value^1^
Rupture			0.350
No	13 (7.78)	57 (34.13)	
Yes	25 (14.97)	72 (43.11)	
Location			0.093
BA	3 (1.80)	5 (2.99)	
ACA A1	0 (0.00)	1 (0.60)	
ICA OPT	4 (2.40)	8 (4.79)	
MCA	11 (6.59)	41 (24.55)	
ACoA	10 (5.99)	20 (11.98)	
ACoP	5 (2.99)	43 (25.75)	
PCA	1 (0.60)	0 (0.00)	
Pericallosal	3 (1.80)	8 (4.79)	
PICA	1 (0.60)	3 (1.80)	
Multiple			0.451
No	17 (10.18)	48 (28.74)	
Yes	21 (12.57)	81 (48.50)	
Size			<0.001
Small	22 (13.17)	113 (67.66)	
Large	11 (6.59)	16 (9.58)	
Giant	5 (2.99)	0 (0.00)	
No. of Aneurysms Clipped in the Same Operation			1.00
1	26 (15.57)	88 (52.69)	
2	11 (6.59)	35 (20.96)	
≥3	1 (0.60)	6 (3.59)	

Source: the SAME of the ISCMSP - Discipline of Neurosurgery.

1 Fisher’s Exact Test, at 5% significance level.

Legend: BA - basilar artery; ACA A1 - segment A1 of anterior cerebral artery; ICA OPT - ophthalmic segment of internal carotid artery; MCA - middle cerebral artery; ACoA - anterior communicating artery; ACoP - communicating segment of internal carotid artery; PCA - posterior cerebral artery; Pericallosal - pericallosal artery; PICA - posterior inferior cerebellar artery
